# Health policy and systems research capacity development to support maternal, new-born, child and adolescent health in West and Central Africa

**DOI:** 10.4314/gmj.v56i3s.2

**Published:** 2022-09

**Authors:** Irene A Agyepong, Edwine Barasa, Kabir Sheikh, Uta Lehmann, Lucy Gilson, Yawa Dahoui, Sue Godt, Issiaka Sombie

**Affiliations:** 1 Ghana College of Physicians and Surgeons, 54 Independence Avenue, Accra. PMB 429, Ministries, Accra / Dodowa Health Research Center, P.O. Box DD1, Dodowa; 2 Health Economics Research Unit, KEMRI Wellcome Trust Research Programme, Nairobi, Kenya; 3 Alliance for Health Policy and Systems Research, World Health Organization, Geneva; 4 Director, School of Public Health, University of the Western Cape; 5 University of Cape Town; 6 Independent translator and interpreter; 7 Retired, independent consultant; 8 West Africa Health Organization, Bobo-Dioulasso, Burkina Faso

**Keywords:** Capacity, Networking, Health Systems, Maternal New-born Child and Adolescent Health (MNCAH), West Africa, Cameroon

## Abstract

**Objectives:**

To examine how and why a South-South capacity development and networking program for leadership, research, practice and advocacy on maternal new-born, child and adolescent health and health policy and systems strengthening in West Africa and Cameroon worked and identify lessons for low- and middle-income countries.

**Design:**

Single qualitative case study drawing on data from document review, observations, key informant interviews and a deliberative workshop. Ethics approval for primary data collection was obtained from the Ghana Health Service Ethical Review Committee (GHS-ERC 012/10/18).

**Setting:**

West Africa and Cameroon

**Participants:**

Researchers, policy and programme managers and frontline health workers

**Interventions:**

Networking and capacity development

**Results:**

The programme made good progress in implementing many but not all planned capacity development and networking activities. The opportunity to network with other organisations and individuals and across countries, disciplines, and languages as well as to learn, to develop skills, and obtain mentorship support, were considered valuable benefits of the partnership. Human and financial resource constraints meant that not all planned interventions could be implemented.

**Conclusions:**

Lessons for health policy and systems research capacity building in LMIC include the potential of South-South partnerships, the need for dedicated resources, the potential of Sub-regional health organizations to support capacity building and recognition that each effort builds on preceding efforts of others, and that it is important to explore and understand where the energy and momentum for change lies.

**Funding:**

The work described here has been funded by IDRC Canada under research grant # 108237 “West and Central African partnership for maternal, new-born, child and adolescent health research.”

## Introduction

Sub-Saharan Africa did not attain the health-related MDG goals and has carried the agenda into the SDG. Despite substantial progress, it remains the region of the world with the highest maternal and neonatal mortality rates.^1^ It also has persisting health system weaknesses. Strong health systems are essential to support the delivery of programs and interventions to improve population health. 2,3 Building such systems requires strong research as well as leadership, policy and program development and implementation capacity.^4^ Franzen et al^5^, in their review of the qualitative literature on health research capacity development in low- and middle-income countries (LMIC) between 2000 and 2013, identified the importance of systems approaches, demand for stronger links between research, policy and practice and the effect of power relations on capacity development. They categorised the modalities for capacity development into four main groups vertical research projects, centres of excellence, north-south partnerships, and networks, with strengths and weaknesses in each of these approaches.

Following a yearlong sub-regional consultative process in 2015 to explore how to strengthen health policy and systems research (HSPR) to better support maternal, new-born, child and adolescent health (MNCAH) improvements in West Africa, one of the things that strongly emerged was the felt need for a sub-regional led and owned network and capacity development approach with links between research, policy, practice and a systems approach. A proposal was therefore developed for a consortium of researchers, decision-makers and implementers embedded in institutions in West Africa and Central Africa (Cameroon) in collaboration with the West Africa Health Organization (WAHO), and researchers in the University of Cape Town and the University of the Western Cape in South Africa to form a South-South capacity development, networking and advocacy partnership for leadership, research, and practice for HPSR strengthening and MNCAH. The goal was to contribute to stronger health systems for improved MNCAH outcomes. This was to be done through multi-level capacity development and networking at individual, organizational and contextual (national and West African sub-regional) level to support context-relevant and effective leadership, research, policy and program decision-making and implementation for Health Systems strengthening and MNCAH improvements. The initiative, which was named the Consortium for mothers, children, adolescents and health policy and systems strengthening (COMCAHPSS) was unique in that in capacity development outputs were as much valued as research and in its focus on South-South led and owned networking, capacity, and advocacy.

### Objectives

In this paper we ask the question: how did this program work (or not), why, and what lessons have emerged for other low and middle-income countries? Specifically, we ask: how appropriate and relevant were the design considerations, such as conceptual framework, governance mechanisms and structures of the program in the light of the problems it set out to help address? What were the key barriers and enablers of the implementation of the program, and what was it able to achieve (or not)? What are the lessons for multi-country networking and capacity strengthening to support health systems strengthening and health outcome improvement in LMIC?

### Conceptual framework

**Figure 1:**
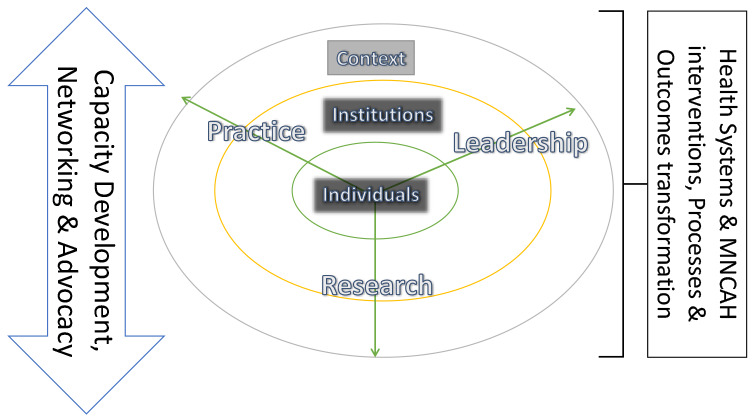
Capacity Development framework

The conceptual framework that guided the interventions of COMCAHPSS drew upon health systems and capacity development frameworks in the literature. (Figure 1)

Health systems were conceptualised as a shelter that protects the population's health. Capacity was conceptualised as the ability to perform and produce desired outcomes and a dynamic process involving complex multi-level relationships within and between organisations, contexts, and individuals. 6,7,8 Individual capacity refers to competencies needed to enable strategic (macro), operational (meso) or team (micro) level health systems leadership, research, and practice, including whether individuals are sufficiently knowledgeable, skilled, experienced, confident, and motivated to adequately perform. It is a function of individual skills and motivation but also of the organisation's capacity in which the individual functions and the context within which the organisation is embedded.

Organisational capacity refers to the ability of organisations to support the required performance of individuals within them. Organisational design, infrastructure, availability, and appropriateness of tools, staffing numbers, skills, and distribution, as well as culture (how things are done around here) and climate (how it feels to work around here) influence organizational capacity.

Contextual capacity is the ability of wider international, national, and sub-national social, economic, historical, political, structural and situational factors and systems to support organisational and individual performance.^9^, ^10^, ^11^ Capacity development, networking and advocacy must occur across practice, leadership and research and also across individual, organisational and contextual levels if they are to translate into health systems strengthening and population health outcome improvements.

## Methods

The study design was a single qualitative case study of the COMCAHPSS program. A case study enabled us to investigate a complex phenomenon within its real-life context.^12^ Social processes are complex, and an appropriate study design should aim to develop concrete, context-dependent knowledge.^13^ A case study was also suited to obtaining the multiple perspectives, experiences and interactions of various stakeholders and social processes. In terms of limitations, a single case study design is analogous to a single experiment, and we cannot assume that its findings are generalisable to all contexts. 14

### Interventions

Because of the intersecting themes of research, practice, and leadership at multiple levels against capacity development, networking and advocacy, the planned interventions when the program started in April 2016 are best described in a grid as in Table 1.

**Table 1:** Planned Program interventions and objectives

	Individual & institutional level		Contextual level
Themes	Capacity development	Networking	Collaboration with WAHO
**Leadership**	West African bi-annual seminars: Leadership modules leadership mentorship program Support to Pan African DrPH program development and implementation	West African network of emerging leaders in HPSR (WANEL) Communities of practice Global conference e.g., HSG participation support grants for emerging leaders with accepted abstracts Annual partnership meetings	Promotion evidence use for decision making Strengthened collaboration between researchers and decision makers
**Research**	West African bi-annual seminars: HPSR and MNCAH HPSR and MNCAH studies Research supervisor / mentor program Specialist Master's program Peer-reviewed journal publication support		
**Practice**	West African bi-annual seminars: “how to” work with decision makers and implementers Research communication Researcher, media, and civil society engagement		
**Monitoring** **and Evaluation**	Qualitative process documentation and evaluation Quantitative output and impact documentation Realist Evaluation of the program (‘how’ and ‘why’ the program works to produce the documented impacts) External Mid-term evaluation		

Interventions intended but eventually not implemented because of funding constraints are shown in shaded grey. At its inception, the Consortium had 19 partner institutions from 9 countries (Table 2).

**Table 2:** COMCAHPSS consortium partners and countries at inception

Country	Consortium partner
**Benin (Francophone)**	1. Centre for Research in Human Reproduction and Demography (CERRHUD)
**Burkina Faso (Francophone)**	2. WAHO 3. West Africa Health Research Network (WAHRNET) 4. Institut Supérieur des Sciences de la Population (ISSP)
**Cameroun (Bilingual Anglophone/Francophone)**	5. Biotechnology Centre 6. The Centre for the Development of Best Practices in Health (CDBPH) 7. Higher Institute for Growth in HEalth Research for Women ( HIGHER Women)
**Cote d'Ivoire (Francophone)**	8. Université Houphouet Boigny /Ivorian Public Health Association
**Ghana (Anglophone)**	9. University of Ghana (UG) 10. Ghana Health Service Research and Development Division (GHS RDD) 11. Ghana institute of Management and Public Administration (GIMPA) 12. ABANTU for Development 13. African Media and Malaria Research Network (AMMREM)
**Mali (Francophone)**	14. L'Institut National de Recherche en Santé Publique (INRSP)
**Niger (Francophone)**	15. Laboratoire d'Etudes et de Recherche sur les Dynamiques Sociales et le Développement Local (LASDEL)
**Nigeria (Anglophone)**	16. College of Medicine University of Nigeria Enugu Campus (COMUNEC) 17. National Primary Health Care Development Agency (NPHCDA) 18. Health Reform Foundation of Nigeria (HERFON)
**Senegal (Francophone)**	19. Institut de Santé et Développement (ISED)
**South Africa**	20. University of Cape Town 21. University of Western Cape

Data sources were document reviews, observation, key informant interviews (KII) and a deliberative workshop.^15^ Documents reviewed were minutes of meetings, program and workshop reports, the project proposal, and partner reports. Observations were from IA, SG and IS who were “insiders” to the program involved in its design, governance, and implementation. Key informant interviews and the deliberative workshop were conducted in 2018 as part of a mid-term review by a team comprising EB, KS and YD who were “outsiders” to the program in that they were not part of program conceptualisation, implementation, or governance.

KII are useful to obtain information about perceptions of the program, barriers and enablers, relevance of the conceptual framework and experiences of the program programmes from the perspective and experiences of a wide range of programme stakeholders.^16^ The selection of participants for KII was purposive to select stakeholders with in-depth knowledge of the programme. They included implementers, beneficiaries, academics, and policymakers. Ethics approval was obtained from the Ghana Health Service Research Ethics Review Committee ((GHS-ERC 012/10/18). All data collection was done with informed consent and anonymised by removing personal identifiers and using codes.

The deliberative workshop was held with consortium members and the Consortium advisory committee during a consortium partners and Advisory Committee meeting in Accra in 2019. The five-member consortium advisory committee comprised senior and respected research, policy, and practice leaders in West Africa and globally from Burkina Faso, Cameroon, The Gambia, Ghana, and Niger.

### Data Management and Analysis

Documents were analysed manually for themes, commonalities, and contrasts. All KII were recorded with informed consent, transcribed into Microsoft word, and imported into NVIVO software for coding and analysis using a framework approach.^17^

## Results

A total of twenty-six (26) KII were conducted. (Table 3)

**Table 3:** Background characteristics of mid-term review Key informants

Country	No. interviewed	Category	No. interviewed
**Ghana**	9	Academic/Researcher	7
		Policy Maker/Implementor	2
**Niger**	7	Academic/Researcher	7
		Policy Maker/Implementor	-
**Nigeria**	3	Academic/Researcher	1
		Policy Maker/Implementor	2
**Cote** **d'Ivoire**	1	Academic/Researcher	1
		Policy Maker/Implementor	-
**Benin**	2	Academic/Researcher	-
		Policy Maker/Implementor	2
**Senegal**	2	Academic/Researcher	1
		Policy Maker/Implementor	2
**Burkina** **Faso**	2	Academic/Researcher	1
		Policy Maker/Implementor	1
**Total**	**26**		**26**

### Perception of the problems of HPS and MNCAH in West Africa

Respondents in the mid-term review, emphasized that poor MNCAH outcomes in West Africa were partly driven by weak health systems. The 2014 West African Ebola epidemic was a stark revelation of the health system challenges in the sub-region and a wakeup call.

*“... in 2014 when we had an Ebola crisis that revealed the vulnerability of health systems across the sub region....”* IDI 15

They also felt that the issue was not uniform weaknesses but rather varying levels across and within countries, with a mix of strengths and weaknesses.

“*.... there is a lot of variability between countries. I would say [West Africa] is a relatively fragile sub-region compared to the rest of the world, but it is not uniform fragility...Niger is fragile within Francophone West Africa, but they are actually strong in health systems and anthropology research because of LASDEL. So, it's a funny mix.”* IDI 11

Health research capacity was also seen as variable with a mix of strengths and weaknesses, and gaps were related to numbers and skills. Inadequate numbers meant high workloads that sometimes worsened the ability to perform even when people were skilled.

“*The lack of human resources... if they were much more, they could do better ... most of the researchers ... are also professors at the university which is a good thing on its own. But because of the time constraint, they are not able to fully devote themselves, and then even when they have something done, they are not able to publicize or disseminate the information ....people don't get to know what they have done”* IDI 1

Also seen as challenging was the low prioritisation of research funding by the government and the dependence on external funding, which meant that priorities were sometimes externally driven and perhaps not necessarily the most urgent local priorities.

English was seen as the dominant language of international research and publication. Francophone researchers felt disadvantaged by their inability to access the literature and to publish in English and saw it as limiting their publication output and reach. One of the perceived benefits of COMCAHPSS was the opportunity for Francophone researchers to access research and publish their work in English.

Developing and sustaining a culture and capacity for research uptake and evidence informed policy making is an important part of health policy and systems research.^18^,^19^ Program participants perceived weaknesses and strengths in this area within and across countries.

*“...we have the data, how many of us are using the data to inform decisions?”* IDI 3

“*...The other thing is that they have a lot of research available, but they are not used effectively to be able to bring the evidence first in the formulation of the policy.”* IDI 2

Strengths and weaknesses were not seen as uniform between countries across the sub-region. For example, in the context of West Africa, Ghana was perceived to be relatively advanced in the use of evidence to inform formulation of government interventions and good relationships between the researchers, the Ministry of Health and its agencies. However, within Ghana, there were perceptions of gaps in use of research evidence to inform policy and program formulation and implementation and a need for greater utilization of routine and research data to inform policies and programs.

There was a strong understanding of the social and political rather than purely technical nature of research uptake efforts and capacity. For example, in Niger, respondents described how they overcame a situation where use of evidence to inform policy was minimal due to strained relationships between government actors and researchers because the government felt the researchers were attacking its shortcomings. Consistent engagement over time and knowledge sharing enhanced trust and collaboration between both parties and created opportunities for increased use of research to inform decision-making.

What the program was able to achieve?

The programme made good progress in implementing many of its planned interventions. However, as observed by a KI in the mid-term review, realistically, like for many capacity development efforts, its full impact will only become apparent in the long term.

*“...the kind of work that you are doing is really a long-term proposition, so you are just at the point where you are just starting to train cohorts, whether they are the implementers, whether they are researchers....”* IDI 5

For most participants, the opportunity to network with other organisations and individuals and across countries, disciplines, and languages as well as the opportunity to learn, develop leadership skills, and obtain mentorship support, were considered valuable benefits of COM-CAHPSS and individual and team capacity building and networking were perhaps the best implemented interventions. This included supporting young researchers to submit abstracts and attend and present at local and international conferences, including the health system global symposiums in 2016 and 2018. This was achieved partly by linking senior mentors in the sub-region with early career researchers for review, guidance, and support in developing their abstracts as part of a process of catalysing and leveraging resources within the region. Beneficiaries of this support appreciated the capacity development objective but also saw it as a networking opportunity and a platform to meet and develop networks with other early career and senior researchers.

A six-week pre-doctoral research capacity development program in Accra followed by a one-week program with the partners in Cape Town also targeted capacity development for 8 early career researchers embedded in partner institutions in the network and was widely appreciated.

As part of longer-term capacity development, 6 of the participants in the 2017 pre-doctoral researcher capacity building program were supported in part or in full for their doctoral level training either through small grants for their research or full PhD training funding, including fees, and living allowances. This involved exploration, advocacy, and dialogue to leverage funding additional to the two PhD training support grants that could be mobilised from the IDRC funding to COMCAHPSS itself. This resulted in WAHO providing a full scholarship to one of the trainees and another being supported to successfully obtain WHO Alliance for Health Policy and Systems Research Health Policy Analysis fellowship program support. 20 Networking with Canadian researchers in the University of Montreal enabled enrollment there with tuition support for the trainee from Niger.

The West African network of emerging leaders (WANEL) was supported with meeting organisation through the COMCAHPSS secretariat, participation in international conferences (39 members over the five years), guidance in network development including links with other partners such as AfHEA and WAHO, mentorship, protocol development and implementation. By the final year of COMCAHPSSS WANEL had been registered as a West African not for profit non-governmental independent professional network of early and mid-career health policy and systems researchers in the West African sub-region with representation on the board of the African Health Economics and Policy Association (AfHEA).^21^

Drawing on the concepts and materials developed through the COMCAHPSS program, a mid-Year school in August 2019 and a New-Year school in February 2020 provided individual and team capacity development for country level communities of practice to co-produce research, interventions, and advocacy for MNCAH. The COMCAHPSS funding could only support one country team from Senegal. However, five additional country teams from Burkina Faso, Cote d'Ivoire, Ghana Niger, and Sierra Leone, after the secretariat were supported to participate through the IDRC funded Women, new-born, Child and Adolescent (WNCAW) program.^22^

The development of a research master's program was not implemented. The Health Leadership Africa or Pan African Doctor of Public Health program concept for leadership training was not implemented either. However, the concept and materials developed with earlier funding from the Rockefeller foundation were built upon and used to inform the health policy management and leadership track in the faculty of Public Health of the Ghana college of Physicians and Surgeons. 23 The planned realist evaluation of the program was scaled back to the qualitative case study of the program presented in this paper.

### Why did the program achieve (or not)

The program achieved for several reasons. Firstly, shared values across the partnership that included working together, solidarity, excellence, and transparency enabled the networking with other organizations and individuals, across countries and disciplines, peer to peer learning as well as mentorship that were considered valuable benefits. Secondly, the flexibility on the part of both implementers and the funder and an open-door policy, made it relatively easy to adapt rapidly to context and emerging observations. For example, funds that were initially budgeted to provide small research grants to early and mid-career researchers at country level were retooled to support the 2017 pre-doctoral research capacity development program and provide added support for doctoral level training as it became clear that the capacity to design and conduct high-quality health systems research needed to be urgently addressed to make small grant funding more effective.

Participants in the mid-term review also mentioned visionary leadership, effective leveraging on pre-existing individual and institutional relationships, a growing interest in health systems research in West and Central Africa and networking across early career and senior researchers, decision makers and practitioners as important reasons COMCAHPSS achieved what it did.

Program participants appreciated the importance of a multi-level capacity development framework targeting individual, organisational, and contextual levels. However, many felt that in practice, it was more challenging to implement and evaluate the impact of interventions to develop organisational and contextual capacity compared to Individual capacity.

*“In my opinion, individuals have in a way built their competencies whether in research or in policy or in practice. However, it is difficult to link what has been done... to change within an organization... it will be difficult for me to say COMCAHPSS has influenced discussions around the ministry or the university .... It is difficult to say whether the individuals can influence organizational change or discussions, ....”* IDI 4

Several reasons were felt to account for this. Firstly, individuals regularly change employment which limits their effectiveness in diffusing capacity within targeted organisations. Secondly, for organizational capacity development to occur, a critical mass of individuals within an organisation must be targeted. However, the program financial constraints meant that only a few individuals could benefit from the capacity development efforts. Thirdly, there was the not-always-easy task of getting buy-in from the organisational leadership and ensuring alignment of capacity development initiatives with their objectives. Finally, the implemented COMCAHPSS program interventions focused more on individual capacity building and networking than organisational capacity building.

The initial cost of what it would take for the partners to accomplish the ambitious set of COMCAHPSS objectives and interventions was almost three times the five-year grant support of approximately USD 800,000 provided by IDRC Canada. In the resource-constrained context of West Africa, almost no funding was available from the countries. Rather than dismantle the holistic vision of the proposal, a decision was taken to adopt a stepped implementation approach while conducting parallel searches for co-funding. The interventions prioritised to use COMCAHPSS funding for in this stepped approach were to protect the lean secretariat of one full-time bilingual research and administrative assistant, one part-time post-doctoral researcher and one senior researcher, support to WANEL, maintenance of the advisory committee, annual partner meetings and the individual capacity building interventions.

The funding constraint meant that in the end some planned interventions were not implemented at all, or full implementation was considerably delayed.

“*We were not able to do the summer school because we went through the whole process, we designed the curriculum, we met several times to look at it, discuss it, fix dates and all and the adverts was in there, people applied but they really had no funding. So, then it had to be postponed”* IDI 4

The failure to develop the shared open-access research master's curriculum was not implemented for similar reasons. Concerning the Pan African DrPH Health Leadership Africa concept, despite COMCAPHSS building on the work started with a grant from the Rockefeller Foundation as already mentioned, convened a potential partner meeting, and further developed some of the ideas into an open access training module made available online in French and English, partners felt they did not have the capacity to move forward to implement the program without extra human resource and funding support. The concepts and training materials were, however, incorporated into the curriculum of the Ghana College of Physicians and surgeons Public Health program health policy management and leadership track.

Some mid-term respondents felt that the resistance to the introduction of the DrPH Health Leadership Africa was not only because of the human and financial resource constraints but partly because academic faculty were not familiar with the concept, which was somewhat different from the traditional masters and PhD degrees they already run and did not quite understand where it fitted into the curriculum for public health. They were apprehensive about their capacity to deliver the curriculum.

There were no plans to drop any of the original program partners (table 2). However, Inclusiveness requires the investment of time to maintain communication, respond to communication, convene partners, remind people, support processes and adequate provision of resources for implementation. Time allocated to activities was considered the greatest cost of the partnership. The secretariat was lean relative to the breadth of the partnership, all estimated resources needed were unavailable and the success of efforts to find extra resources was uncertain. When some partners were inactive, it was easy for the secretariat to just let it go. In the end, four partners from Mali, Ghana, Senegal, and Burkina Faso did not actively engage after the program's inception. Ghana had multiple partners, and the non-engagement of one partner did not appear to make a difference. In Senegal, largely thanks to the representative on the advisory committee, another partner replaced the non-active partner. In Mali there was only one partner, no replacement emerged, and the country was lost to the program.

Despite this, most respondents in the mid-term review felt that COMCAHPSS had strong and transparent governance arrangements, and this helped the program. However, a few expressed concerns about the loss of some initial partners and felt that they were not sufficiently aware of the governance arrangements, and even when they were aware of them, were not clear as to their roles.

## Discussion

The observation by the Global Forum on health research almost two decades ago in the 10/90 report on health research 2003–2004 of the inequity gap between LMIC and high-income countries (HIC) in health research remains current. That the search for effective interventions continues is manifest in the many efforts and approaches to bridge the gap.^24^,^25^ There are several lessons from our experience in the COMCAHPSS program that can contribute to the increasing global experience and literature. They can be summarized as: South-South partnerships have potential to contribute, dedicated resources are needed, Sub-regional health organizations also have the potential to contribute, each effort builds on preceding efforts, and it is important to explore and understand where the energy and momentum for change lies.

### South-South partnerships

South-South partnerships have not been adequately evaluated and documented as an approach to health systems research capacity building. Unique in the COMCAHPSS effort has been the predominantly South-South networking and partnerships. It is an approach that has dealt with some of the long standing and continuing concerns about power relations such as who sets the agenda, who leads research and whose priorities matter^26^. The approach has required understanding of partner strengths and weaknesses, and a strategic mix of partners with varying levels of capacity and a commitment to cross-learning for mutual support and development. Efforts have also been needed to overcome barriers to equitable collaboration posed in West Africa by the Anglophone Francophone official language divide.

### Dedicated Resources

In the context of the resource constraints of LMIC some dedicated resources will continue to be needed for some time to come from external sources. The partnership described in this paper would not have been possible without the funding from IDRC. However, is also important to conduct sustained advocacy to convince national and local governments and legislators to provide some core funding for research capacity building and South-South collaborative efforts if they are to have long-term sustainability.

### Sub-regional health organisations

The potential of sub-regional bodies to support South-South capacity development is perhaps not adequately recognized. The involvement and commitment of the West Africa health organization (WAHO) has been an important contribution to the achievements of COM-CAHPSS. WAHO was launched in 1987 by the heads of state of the 15 member countries of the Economic Community of West African States (ECOWAS), to coordinate health interventions in the sub-region.^27^,*28* There are similar bodies in other LMIC sub-regions whose participation in research capacity building is worth exploring.

Each Effort builds on preceding efforts

An important lesson from COMCAHPSS is that each effort builds on preceding efforts. It is no accident that the secretariat for this effort was hosted in Ghana. The success of preceding efforts over a couple of decades to build health research capacity in Ghana account in part for the research leadership that made the effort possible. Starting in the late eighties, the health sector in Ghana has built up a health research system in the public sector that collaborates and works with academic researchers as well as practitioners and policy makers.^29^,^30^

### Understanding where the energy and momentum for change lies

Identifying, nurturing, and supporting strong, committed, visionary multi-level leadership that is ready to take some risks when needed is critical for health policy systems research capacity building. Nurturing the West African network of emerging leaders (WANEL) into an independent sub-regional professional network holds promise for the future. WANEL identified and built on new talent but also on the talent from emerging West African researchers who had participated in pre-existing efforts by the Institute of Tropical Medicine (ITM) Antwerp emerging voices and the CHEPSAA/CHESAI emerging leaders^31^ capacity building efforts.

Also importantly, given the widespread modality of fragmented project funding and the pressure to meet funder priorities, the more that regional and national partnerships can be nimble and ‘use’ the different funded projects to take forward synergized regional and national plans and priorities, the greater the chance for making progress – given that progress takes time, and most funding is for short periods.

The decision to be creative about the effective use of what was available and to invest efforts to find extra funding, and the results, showed that though these challenges can be frustrating and slow progress, they are not an absolute barrier. Moreover, the efforts to obtain further international competitive grant funding, ended up being in themselves a form of capacity development with learning by doing from failure as well as success.

## Conclusion

There are several lessons for the global community on LMIC research capacity strengthening. Firstly, critical to transformation is identifying, nurturing and supporting strong, committed, visionary multi-level leadership that is ready to take some risks when needed. Secondly is skills strengthening in collaborative problem solving to enable identifying and working with diverse and multi-level actors from global to community level within areas of commonality, no matter how small to start with; and using that as a window of opportunity to widen the circle of influence. Thirdly is a pragmatic approach that focuses on problems and contextually relevant and feasible solutions no matter how mundane they look, rather than chasing after prestigious, attractive, and sometimes very well-funded ‘solutions looking for a problem’. Fourthly is the need for perseverance and staying power that is not easily daunted. Finally, sufficient financial support for an integrated and complex set of activities over the medium to long term is important and should not be lost sight of. However, though it hampers and slows down, it only becomes an absolute barrier to transformation if we throw up our hands in despair.

## References

[1] Maternal and new-born health in low- and middle-income countries: A brief assessment of mortality, coverage, and policies. Countdown to 2030 for Women's, Children's and Adolescent's Health.

[2] WHO (2007). Everybody's Business: Strengthening Health Systems to Improve Health Outcomes, WHO's Framework for Action. Edited by WHO.

[3] Bennett S, Paina L, Kim C (2010). What must be done to enhance capacity for Health Systems Research? Background paper for the global symposium on health systems research.

[4] WHO (2012). Changing Mindsets - Strategy on Health Policy and Systems Research.

[5] WHO, UNICEF, World Bank and United Nations (2015). Trends in Maternal Mortality: 1990 to 2015 Estimates by WHO, UNICEF, UNFPA, World Bank Group and the United Nations Population Division.

[6] Franzen SRP, Chandler C, Lang T (2017). Health research capacity development in low- and middle-income countries: reality or rhetoric? A systematic meta-narrative review of the qualitative literature. BMJ Open.

[7] Agyepong IA, Kwamie A, Frimpong E (2017). Spanning Maternal Newborn and Child Health (MNCH) and Health Policy and Systems (HPS) Boundaries: Conducive and Limiting health system factors to improving MNCH outcomes in West Africa. Health Res Policy Syst.

[8] Potter C, Brough R (2004). Systemic Capacity development: a hierarchy of needs. Health Policy and Planning.

[9] LaFond AK, Brown L, Macintyre K Mapping Capacity in the Health Sector: a conceptual framework January 2002. International Journal of Health Planning and Management.

[10] Potter C, Brough R (2004). Systemic Capacity development: A Hierarchy of Needs. Health Policy and Planning.

[11] UNDP (2006). Capacity Development Practice Note.

[12] Brown L, Lafond A, Macintyre K (2001). Measuring capacity development.

[13] Yin R K (2003). Case Study Research: Design and Methods.

[14] de Lange D E, Flyvbjerg B, Norman K, Denzin Y S L (2011). ‘Case Study’. The Sage Handbook for Qualitative Research.

[15] Yin R K (1999). ‘Enhancing the Quality of Case Studies in Health Services Research’. Health Serv Res.

[16] COMCAHPSS midterm review final report (2019). https://www.wahpscon.org/comcahpss-reports/.

[17] Kvale S (1996). Interviews: An introduction to qualitative research interviewing.

[18] Richie J, Spencer L, Alan B, Robert G B (1994). ‘Qualitative data analysis for applied policy research’. Analysing qualitative data.

[19] (2020). Not Just a Journal Club - It's Where the Magic Happens”: Knowledge Mobilization through Co-Production for Health System Development in the Western Cape Province, South Africa. Int J Health Policy Manag.

[20] Gilson L, Barasa E, Brady L (2021). Collective sense-making for action: researchers and decision makers working collaboratively to strengthen health systems. BMJ.

[21] Final technical report IDRC project # 108237.

[22] Agyepong IA, Godt S, Sombie I (2021). Strengthening capacities and resource allocation for co-production of health research in low- and middle-income countries. BMJ.

[23] Agyepong IA, Lehman U, Rutembemberwa E (2018). Strategic leadership capacity building for sub-Saharan African health systems and public health governance: a multi-country assessment of essential competencies and optimal design for a Pan African DrPH. Health Policy and Planning.

[24] Fosci M, Loffreda L, Velten L, Johnson R (2019). Research Capacity Strengthening in LMIC. Rapid Evidence Assessment Prepared for DfID. June 2019. Report commissioned by the UK Department for International Development.

[25] Käser M, Maure C, Halpaap BMM (2016). Research Capacity Strengthening in Low- and Middle-Income Countries – An Evaluation of the WHO/TDR Career Development Fellowship Programme. PLoS Negl Trop Dis.

[26] White MT (2007). A right to benefit from international research: a new approach to capacity building in less developed countries. Account Res.

[27] WAHO (2020). Who we are.

[28] WAHO (1987). Protocol A/P2/7/87.

[29] World Health Organization Alliance for Health Policy and Systems Research. 2021. Health Policy and Systems Research in Ghana: Current trends and key lessons on how to improve the use of evidence in health policy. Technical brief.

[30] Gyapong J, Ofori-Adjei D Capacity development for Relevant Health Research in Developing Countries.

[31] Nxumalo N (2015). Emerging Leaders in Health Policy and Systems Research & Analysis in Africa Developing the practices of HPSR+A leadership.

